# Perturbation and restoration of the fathead minnow gut microbiome after low-level triclosan exposure

**DOI:** 10.1186/s40168-015-0069-6

**Published:** 2015-03-03

**Authors:** Adrienne B Narrowe, Munira Albuthi-Lantz, Erin P Smith, Kimberly J Bower, Timberley M Roane, Alan M Vajda, Christopher S Miller

**Affiliations:** Department of Integrative Biology, University of Colorado Denver, Campus Box 171, PO Box 173364, Denver, CO 80217 USA; Paul G. Allen School for Global Animal Health, College of Veterinary Medicine, Washington State University, Pullman, WA 99164 USA; School of Molecular Biosciences, College of Veterinary Medicine, Washington State University, Pullman, WA 99164 USA

**Keywords:** Triclosan, Fathead minnow, 16S, Gut microbiome, Denitrification

## Abstract

**Background:**

Triclosan is a widely used antimicrobial compound and emerging environmental contaminant. Although the role of the gut microbiome in health and disease is increasingly well established, the interaction between environmental contaminants and host microbiome is largely unexplored, with unknown consequences for host health. This study examined the effects of low, environmentally relevant levels of triclosan exposure on the fish gut microbiome. Developing fathead minnows (*Pimephales promelas*) were exposed to two low levels of triclosan over a 7-day exposure. Fish gastrointestinal tracts from exposed and control fish were harvested at four time points: immediately preceding and following the 7-day exposure and after 1 and 2 weeks of depuration.

**Results:**

A total of 103 fish gut bacterial communities were characterized by high-throughput sequencing and analysis of the V3-V4 region of the 16S rRNA gene. By measures of both alpha and beta diversity, gut microbial communities were significantly differentiated by exposure history immediately following triclosan exposure. After 2 weeks of depuration, these differences disappear. Independent of exposure history, communities were also significantly structured by time. This first detailed census of the fathead minnow gut microbiome shows a bacterial community that is similar in composition to those of zebrafish and other freshwater fish. Among the triclosan-resilient members of this host-associated community are taxa associated with denitrification in wastewater treatment, taxa potentially able to degrade triclosan, and taxa from an unstudied host-associated candidate division.

**Conclusions:**

The fathead minnow gut microbiome is rapidly and significantly altered by exposure to low, environmentally relevant levels of triclosan, yet largely recovers from this short-term perturbation over an equivalently brief time span. These results suggest that even low-level environmental exposure to a common antimicrobial compound can induce significant short-term changes to the gut microbiome, followed by restoration, demonstrating both the sensitivity and resilience of the gut flora to challenges by environmental toxicants. This short-term disruption in a developing organism may have important long-term consequences for host health. The identification of multiple taxa not often reported in the fish gut suggests that microbial nitrogen metabolism in the fish gut may be more complex than previously appreciated.

**Electronic supplementary material:**

The online version of this article (doi:10.1186/s40168-015-0069-6) contains supplementary material, which is available to authorized users.

## Background

The importance of host-associated gut microbiota to the normal development and overall health of the host organism is well-established and increasingly appreciated [1-3]. The gut microbiome has been shown to be both stable over the long-term [4] and vulnerable to disruption [5] which may have long-term implications for host health [6,7]. Studies on the chemical disruption of host-associated microbiota have generally been concerned with the clinical use of antibiotics [6,8,9], the intentional exposure to personal care products [10], or have focused on the effect of antimicrobials on specific taxa [11]. Less well-characterized are the challenges to the healthy host-associated microbiome from common contaminants, including antimicrobial compounds, particularly at low, but environmentally relevant levels. Even short-duration, low-concentration exposures may alter the gut flora during developmentally important windows.

One such emerging contaminant is triclosan (5-chloro-2-(2,4-dichlorophenoxy)phenol), a chlorinated aromatic compound that has been used as an antimicrobial since the 1960s [12]. Triclosan has limited clinical application, but is frequently found as a component of personal care products and household products [13]. Triclosan is ubiquitous, enters the wastewater system, persists through the wastewater treatment process [14], and has been detected in surface waters at concentrations up to 2.3 μg L^−1^ [15]. Triclosan has been associated with adverse physiological and developmental outcomes [16-18], is photo-degraded to produce dioxins [13], and is potentially an endocrine disruptor in fish [19]. Recently, the United States Food and Drug Administration has reopened discussion of the regulation of triclosan [20], and the state of Minnesota has banned the sale of consumer products containing this compound [21]. Despite this increased interest in triclosan, nothing is known about indirect effects on host health, either due to the triclosan-mediated alteration of the microbiome, or due to the microbially mediated transformation of triclosan.

We hypothesized that low, environmentally relevant levels of triclosan exposure are sufficient to disrupt the fish gut microbiome. To test this hypothesis, we exposed larval fathead minnows (*Pimephales promelas)* to two levels of triclosan (100 and 1,000 ng L^−1^) in a controlled laboratory experiment and used high-throughput 16S rDNA sequencing to profile gut microbial communities before exposure, immediately following an acute exposure window, and after depuration. We also sought to provide a census of the gut bacterial community of the untreated juvenile fathead minnow, an important environmental toxicology model organism [22].

## Results

### 16S V3-V4 variable region sequencing of fish gut microbiomes

**Figure 1:**
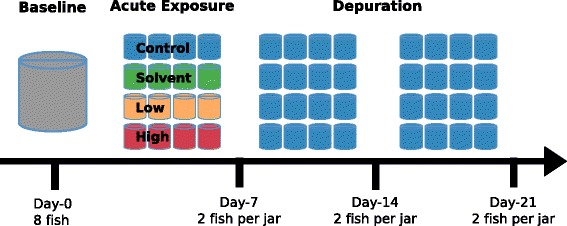
**Experimental design of triclosan exposure experiment.** Fathead minnows were exposed to low (100 ng L^−1^) or high (1,000 ng L^−1^) levels of triclosan, solvent (methanol), or control (no extra chemicals) solutions for 7 days. Multiple fish were housed in each jar, and two fish gastrointestinal tracts were sampled from each jar for microbiome characterization immediately after exposure (day 7) or after 1 and 2 weeks of depuration (day 14 and day 21). Eight fish gastrointestinal tracts were also sampled prior to the acute exposure (day 0).

Using 16S hypervariable region sequencing, we characterized gut microbiomes of fathead minnows in a controlled triclosan exposure experiment. Fish were divided into four exposure groups representing low and high triclosan exposure levels and unexposed and solvent-only control groups (Figure 1). A total of 103 microbiomes were characterized, including eight baseline samples (day 0), samples collected immediately after a 7-day exposure window (day 7), and during a depuration period (day 14 and day 21). At each time point, eight fish were collected for each exposure group. Due to a failure in DNA extraction, one fish from the day 14 low exposure group was not sequenced. An additional five samples were sequenced: one technical replicate (repeat PCR, sequencing, and analysis), one mock community constructed of ten bacteria and archaea (Table 1), and three ‘spike-in’ control samples consisting of a combination of sample gDNA and mock community DNA. Dual-indexed 2 × 250 bp Illumina MiSeq sequencing of 16S rDNA region V3-V4 amplicons from all 108 fish gut and technical samples produced 17,068,840 valid read pairs (Additional file 1: Table S1). All samples sequenced were included in all subsequent analyses. After merging reads, operational taxonomic unit (OTU) picking at 97% identity, and removal of chimeric sequences (see the ‘Methods’ section), we identified 695 OTUs, 10 of which corresponded to the 10 organisms spiked in as members of a mock community. These 695 OTUs were represented by 11,118,352 merged read pairs. Of these 695 OTUs, 94 (13.5%) passed our filtering procedure and accounted for 9,012,689 merged read pairs (81.1% of pre-filtering reads). To confirm that our strict filtering procedure did not alter the conclusions presented here, we also performed all analyses presented below on the unfiltered OTU table and found similar community composition and dynamics for all time and exposure conditions (Additional file 2: Figure S2 and Additional file 3: Figure S3).

**Table 1:** **Composition and accuracy of identification of mock community 16S rDNA sequences**

**OTU ID**	**Pipeline assigned taxonomy**	**Mock community ID (ATCC or GenBank)**
OTU13	k__Bacteria; p__Proteobacteria; c__Gammaproteobacteria; o__Alteromonadales; f__Shewanellaceae; g__Shewanella; s__	*Shewanella amazonenesis* SB2B ATCC: BAA-1098
OTU32	k__Bacteria; p__Firmicutes; c__Bacilli; o__Lactobacillales; f__Lactobacillaceae; g__Pediococcus; s__	*Pediococcus pentosaceus* ATCC: 25745
OTU27	k__Bacteria; p__Firmicutes; c__Bacilli; o__Bacillales; f__Bacillaceae; g__Bacillus; s__	Uncharacterized *Bacillus* isolate GenBank ID: KP025972
OTU21	k__Bacteria; p__Firmicutes; c__Bacilli; o__Lactobacillales; f__Lactobacillaceae; g__Lactobacillus; s__zeae	*Lactobacillus casei* ATCC: 334
OTU20	k__Bacteria; p__Firmicutes; c__Bacilli; o__Lactobacillales; f__Streptococcaceae; g__Lactococcus; s__	*Lactococcus lactis* SK11 ATCC: BAA-493 *Lactococcus lactis* IL403
OTU26	k__Bacteria; p__Firmicutes; c__Bacilli; o__Lactobacillales; f__Lactobacillaceae; g__Lactobacillus; s__brevis	*Lactobacillus brevis* ATCC: 367
OTU30	k__Bacteria; p__Proteobacteria; c__Gammaproteobacteria; o__Enterobacteriales; f__Enterobacteriaceae; g__; s__	*Escherichia coli* K12 GenBank ID: U00096
OTU39	k__Bacteria; p__Actinobacteria; c__Actinobacteria; o__Actinomycetales; f__; g__; s__	*Acidothermus cellulolyticus* ATCC: 43068
OTU294	k__Bacteria; p__Proteobacteria; c__Gammaproteobacteria; o__Enterobacteriales; f__Enterobacteriaceae; g__; s__	*Escherichia coli* K12 GenBank ID: U00096
OTU102	k__Archaea; p__Euryarchaeota; c__Halobacteria; o__Halobacteriales; f__Halobacteriaceae; g__Halobacterium; s__	*Halobacterium salinarum* NRC-1 ATCC: 700922

### V3-V4 sequencing of fish samples is accurate and technically reproducible

In order to assess the accuracy and reproducibility of V3-V4 hypervariable region library preparation and sequencing, we sequenced one mock community sample, three mock community spike-in control samples, and one replicate of sample D0C9. Community membership and abundance were highly correlated across replicate samples (Pearson *r* = 0.99 between D0C9 replicates; Additional file 4: Figure S4). We used the mock community sample to guide quality control and filtering of low-level contaminants and spurious OTUs. Our pipeline identified all ten expected mock community V3-V4 sequences at 100% identity (Table 1; Additional file 4: Figure S4) with the following exceptions: the two subspecies of *Lactococcus lactis* were 100% identical over the V3-V4 region surveyed and were collapsed together into OTU20. *Escherichia coli* K12 was represented by two OTUs. The *E. coli* K12 genome contains seven copies of the 16S rRNA gene, which are identical over the V3-V4 region. The second OTU assigned to *E. coli* was substantially less abundant (OTU294: mock community relative abundance 0.005) than the expected OTU (OTU30: mock community relative abundance 0.063) and may represent a contaminant or variant in the culture from which the DNA was acquired.

Our pipeline assigned 37 OTUs to our mock community sample. Of the reads from this sample, 99.89% were assigned to one of the ten expected mock community sequences, which were also the most abundant OTUs. The remaining 27 OTUs accounted for a total of only 103 reads (0.11%; Additional file 4: Figure S4). These 27 OTUs were all found within the fish samples. Although we cannot rule out the unlikely possibility of miscalled multiplexing indices falsely placing reads into the wrong sample, these 27 OTUs are presumed to be low-level PCR cross-contamination occurring during sequencing library preparation, reflecting the sensitivity inherent to deep DNA sequencing. The most abundant non-mock community OTU found in the mock community sample was OTU1, a member of the family *Aeromonadaceae*. OTU1 represented 32% of all reads in the unfiltered OTU table and was the most abundant OTU in the study. Within the mock community, this OTU accounted for only 0.03% of reads; this abundance level was used as the basis for study-wide OTU filtering (see the ‘Methods’ section and Additional file 4: Figure S4). If present in the experimental samples, the mock community OTUs were successfully filtered out as contaminants. In the spike-in samples, the few remaining non-filtered reads (mean *n* = 263) closely reconstructed the expected D0C9 community (Additional file 4: Figure S4; mean pairwise Pearson *r* = 0.85). Thus, we proceeded to estimate fish gut microbial community composition under the assumption that any observed inter-individual differences were not primarily due to technical artifacts.

### The fathead minnow gut microbiome resembles that of other freshwater fish

To our knowledge, we present here the first deep sequencing census of the gut microbiome of the fathead minnow, a common model organism in environmental toxicology studies. The eight co-housed baseline day 0 samples show low inter-individual variation in microbial community composition (mean pairwise Pearson *r* = 0.80), as estimated based on the relative abundance of 16S rRNA gene sequences observed. Among the most abundant taxa inferred in the fathead minnow gut are many bacteria previously observed in other freshwater fish [23-27]: *Aeromonadaceae* (mean abundance: 8.5% baseline, 57.5% day 7 to 21 control), *Shewanellaceae* (mean abundance: 2.2% baseline, 9.1% day 7 to 21 control), as well as *Flavobacteriaceae, Fusobacteriaceae*, *Deefgea*, *Pseudomonadaceae*, *Plesiomonas*, and *Cetobacterium*. Some of these taxa are included in the set of 20 OTUs that were present across at least 95% of the 103 sampled fish across all time points and exposures, representing a potential fathead minnow ‘core’ microbiome (Table 2; Additional file 5: Figure S5). Relative abundances of many of these core OTUs change with fish development during the course of the experiment in the unexposed controls. For example, OTU10 (*Deefgea*), OTU11 (*Pseudomonas*), and OTU6 (*Rhodobacter*) appear to increase in relative abundance from days 7 to 21, while OTU9 (*Pseudoalteromonadaceae*), OTU4 (CK-1C4-19), and OTU482 (*Aeromonadaceae*) decrease. In addition to considering variation at different developmental stages, definitive identification of a core fathead minnow microbiome would require sampling from a variety of habitats and genotypes and in laboratory settings may be affected by environmental conditions such as water chemistry, water recirculation, and diet.

**Table 2:** **Twenty OTUs form a study-wide core fathead minnow gut microbiome**

**OTU IDs**	**Taxonomic assignment**	**Phylum**	**Mean relative abundance**
OTU2	*Acinetobacter* (genus)	Proteobacteria	0.053 ± 0.057
OTU46	*Acinetobacter* (genus)	Proteobacteria	0.017 ± 0.02
OTU392	*Acinetobacter johnsonii* (species)	Proteobacteria	0.004 ± 0.006
OTU1	*Aeromonadaceae* (family)	Proteobacteria	0.37 ± 0.18
OTU482	*Aeromonadaceae* (family)	Proteobacteria	0.08 ± 0.11
OTU600	*Aeromonadaceae* (family)	Proteobacteria	0.02 ± 0.09
OTU14	*Bacteroidaceae* (family)	Bacteroidetes	0.007 ± 0.012
OTU16	Betaproteobacteria (class)	Proteobacteria	0.008 ± 0.013
OTU8	*Cetobacterium somerae* (species)	Fusobacteria	0.03 ± 0.04
OTU4	CK-1C4-19 (candidate division)	CK-1C4-19	0.03 ± 0.06
OTU10	*Deefgea* (genus)	Proteobacteria	0.02 ± 0.04
OTU12	*Flavobacterium* (genus)	Bacteroidetes	0.01 ± 0.01
OTU18	Fusobacteriales (order)	Fusobacteria	0.005 ± 0.007
OTU9	*Pseudoalteromonadaceae* (family)	Proteobacteria	0.03 ± 0.05
OTU11	*Pseudomonas* (genus)	Proteobacteria	0.02 ± 0.03
OTU40	*Pseudomonas alcaligenes* (species)	Proteobacteria	0.002 ± 0.003
OTU6	*Rhodobacter* (genus)	Proteobacteria	0.03 ± 0.06
OTU7	*Shewanella* (genus)	Proteobacteria	0.1 ± 0.06
OTU760	*Shewanella* (genus)	Proteobacteria	0.001 ± 0.001
OTU5	*Verrucomicrobiaceae* (family)	Verrucomicrobia	0.03 ± 0.04

Mean relative abundance from 103 samples collected across four time points and five exposure categories.

### Gut community composition changes over time

**Figure 2:**
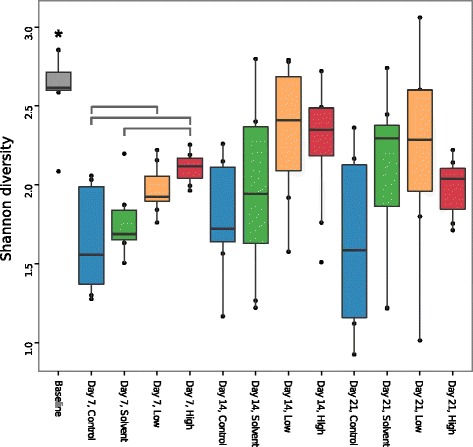
**Alpha diversity pattern with time and triclosan exposure.** Shannon diversity measures are plotted as interquartile range with median for each triclosan exposure class and time point grouping. There is an initial drop in alpha diversity at the beginning of the study. Baseline samples are significantly more diverse than all other time points (denoted by asterisk; Tukey’s HSD, adjusted *P* ≤ 0.05). Within day 7, square brackets denote that the control samples differ significantly from low exposure (*P* = 0.026; estimated difference in mean: 0.327; 95% CI: 0.031 to 0.623) and high exposure samples (*P* = 0.001; estimated difference in mean: 0.464; 95% CI: 0.167 to 0.760) and the solvent samples differ significantly from the high exposure samples (*P* = 0.017; estimated difference in mean: −0.348; 95% CI: −0.644 to −0.052) For each boxplot, *n* = 8 except day 14, low (*n* = 7).

We calculated measures of alpha (within sample) diversity and beta (between samples) diversity among gut microbiomes. Alpha diversity, as measured by the Shannon diversity index, decreased sharply for all exposure groups from baseline (arrival from rearing facility) to day 7 (Figure 2). Two-way ANOVA considering the effects of time point and triclosan exposure group on mean Shannon diversity index indicated no interaction effect (*p* = 0.67), so we considered each main effect separately. Using Tukey’s honest significant difference (HSD) test for multiple comparisons (Figure 2), the baseline samples differ significantly from all other time points (*P* ≤ 0.012). At day 7 only, the control samples differed significantly from both the low and high samples (*P* = 0.026, *P* = 0.001) and the solvent samples differed from the high samples (*P* = 0.017) with alpha diversity increasing in association with triclosan exposure. Within the later time points, there was no significant difference in the mean Shannon diversity index associated with triclosan exposure. The same analyses applied to the unfiltered OTU table produced the same set of significant pairwise differences in alpha diversity.

**Figure 3:**
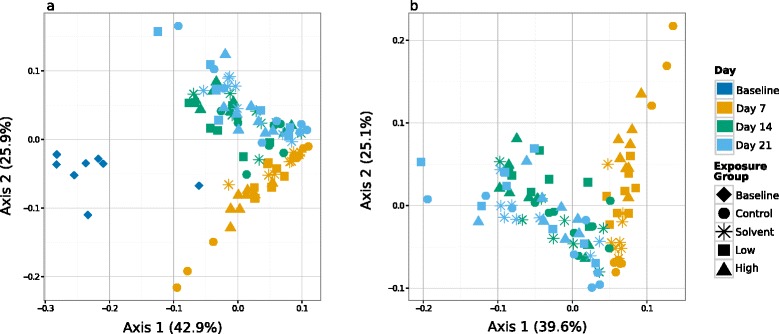
**Fathead minnow gut microbial communities are dynamic over time.** Principle coordinates analyses of weighted UniFrac distances show that gut communities cluster by time point. **(a)** Baseline samples (day 0) are distinct from samples collected at later time points. Samples from day 7 have a unique community structure that differentiates them from days 14 and 21 samples, which are largely indistinguishable from each other. **(b)** PCoA plot of weighted UniFrac distances calculated excluding the highly divergent baseline samples shows a similar divergence of day 7 samples from day 14 and day 21 samples.

For beta diversity, a weighted UniFrac distance matrix [28] among all samples was calculated as input to principle coordinates analysis (PCoA) and as the basis of statistical tests described below. We used the weighted UniFrac metric, as our experimental design was of a closed microbial system, using sterilized materials including water and commercially prepared food (see the ‘Methods’ section): we expect changes in diversity among samples to be driven more by changes in relative abundance than by changes in community membership due to immigration. PCoA of weighted UniFrac distances separated samples primarily according to time point, with 68.8% of variation among samples explained by the first two axes (Figure 3a). PERMANOVA analysis [29] showed significant effects of both time (*F* = 18.4, *df* = 2, *P* ≤ 0.001) and triclosan exposure (*F* = 13.6, *df* = 4, *P* ≤ 0.001), with no interaction effect between the two factors (*F* = 1.2, *df* = 6, *P* = 0.238). Baseline samples were most distant from all other samples. To ensure that the time and exposure effects across the entire experiment were not solely due to the divergence of baseline samples from all other samples, we repeated the principle coordinates analysis and the PERMANOVA after removing the baseline samples (Figure 3b). Again, the samples clustered according to time (*F* = 20.4924, *df* = 2, *P* ≤ 0.001) and triclosan exposure (*F* = 2.2077, *df* = 3, *P* = 0.024), with no significant interaction effect (*F* = 1.3072, *df* = 6, *P* ≤ 0.170). This time signal is not an artifact of the combination of distance and ordination methods. The use of non-phylogenetically based distance methods (Canberra, Morisita) [30] and alternate ordinations generated the same groupings of samples, organized most strongly by time (Additional file 6: Figure S6).

### Environmental exposure to triclosan alters fish gut microbial community composition

**Figure 4:**
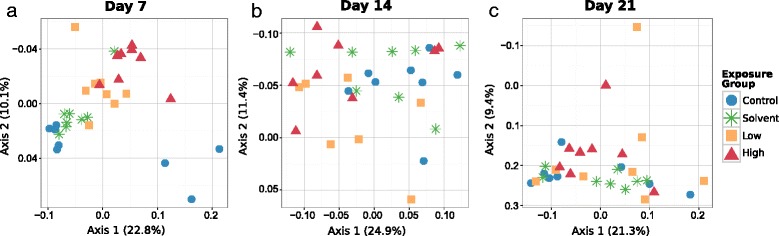
**Triclosan exposure shifts gut microbial community structure. (a)** Samples cluster by exposure level immediately following acute triclosan exposure (day 7). After 1 week **(b)** and two weeks **(c)** of unexposed recovery (day 14 and day 21), samples are no longer separated by triclosan exposure history. Multi-response permutation procedure (MRPP) significance testing using the weighted UniFrac distance matrices shows that clustering of exposed and unexposed samples is highly non-random at day 7 (*A* = 0.194, *P* = 0.0003), but is no longer defined by exposure on day 14 (*A* = 0.056 *P* = 0.043) and day 21 (*A* = 0.003, *P* = 0.385).

In order to test whether environmental exposure to triclosan alters fathead minnow gut microbiome composition independently of time, we evaluated each time point separately. Immediately following the triclosan exposure period (day 7), samples cluster visibly by triclosan exposure (Figure 4a). A secondary cluster of control samples (D7C32, D7C41, D7C42) is driven by the retention of OTUs from the *Verrucomicrobiaceae* and CK-1C4-19 which were present in the baseline (day 0) samples, but reduced in the other day 7 control samples (Additional file 7: Figure S1). At day 7, microbiomes exposed to high levels of triclosan were, on average, more distant from control microbiomes than those exposed to low levels of triclosan. Visual clustering by triclosan exposure disappears over the 2-week post-exposure depuration. Although samples are non-randomly distributed by exposure status at day 14 (Figure 4b), at 2 weeks following exposure (day 21), microbiomes are indistinguishable by exposure type (Figure 4c). We confirmed the significance of the clustering by exposure type using the multi-response permutation procedure (MRPP) [31] on the weighted UniFrac distance matrix. This procedure compares the average observed within-exposure group distance against the within-group distance of randomly permuted groups. The *P* values associated with exposure effect increase from 0.0003 at day 7 to 0.043 at day 14 to 0.385 at day 21 (Figure 4). The use of Canberra and Morisita distance metrics as noted above confirmed (both via ordination and via MRPP) that the observed clustering by triclosan exposure status at day 7 was not an artifact of the choice of distance and ordination metrics (Additional file 8: Figure S7).

### Differential response to time and triclosan exposure can be seen for individual taxa

**Figure 5:**
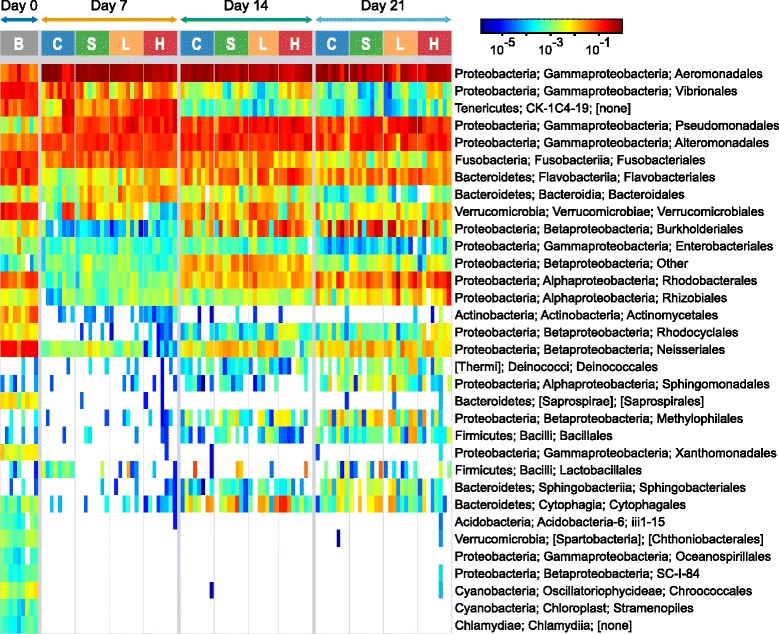
**Order-level relative abundances of individual fish microbiomes.** Relative abundances of OTUs are summarized at the order level for each fish in the exposure experiment. Taxa (rows) were clustered and ordered by single-linkage hierarchical clustering based on Euclidean distances of day 7 cells. White cells in the heat map indicate no OTUs assigned to an order. Taxonomic assignment is by the RDP classifier trained on the Greengenes database, and each row is presented as phylum, class, order. Columns are *B* baseline, *C* control, *S* solvent, *L* low triclosan exposure history, and *H* high triclosan exposure history. *Scale bar* fractional relative abundance.

**Figure 6:**
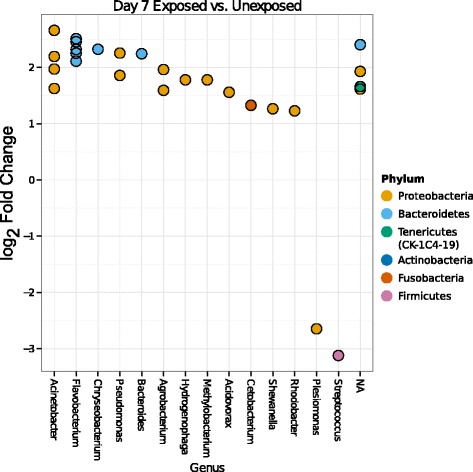
**Significant changes in abundance of selected gut microbiome OTUs following triclosan exposure.** OTUs were identified as significantly differentially abundant (adjusted *P* ≤ 0.005) by exposure status (exposed [high and low] vs. unexposed [control and solvent]) at day 7 via DESeq2 [32]. Genus-level assignments are presented where available.

**Figure 7:**
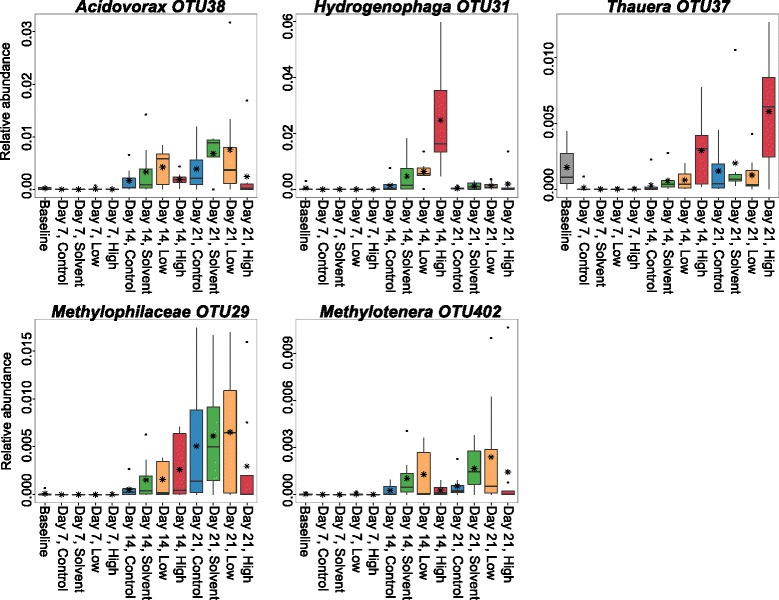
**Denitrification-associated taxa increase in relative abundance following methanol and triclosan exposure.** Shown are relative abundance trends for selected OTUs related to organisms known to be methylotrophic denitrifiers. Boxplots of relative abundance include interquartile range, with median plotted as horizontal line and mean plotted as asterisk. For all boxes, *n* = 8 samples except for day 14, low where *n* = 7 samples. Note differing *y*-axis scales. Significant pairwise comparisons are highlighted in Additional file 11: Table S4.

Examining the community at the order level (Figure 5) identifies specific taxa that contribute to temporal and triclosan exposure-associated changes in overall microbial community structure (Figures 2, 3, and 4). Housing conditions were designed to minimize the chance that changes to microbial community membership could result from newly introduced taxa after the initiation of this study, as all housing materials and water were sterilized. There were no new bacterial orders present in days 7 to 21 that were not present in at least one baseline (day 0) sample. At the OTU level, there were five OTUs present in days 7 to 21 that were never detected in a baseline sample, though each of these OTUs had at least one other closely related OTU in at least one baseline sample at the family or genus level. However, many orders present in day 0 were lost or fell below the level of detection at subsequent sampling time points. Below the order level, 28 individual OTUs were significantly differentially abundant (DESeq2 [32]; adjusted *P* < =0.005) between unexposed samples (control and solvent) and exposed (low and high) samples at day 7 (Figure 6 and Additional file 9: Table S2). These OTUs include but are not limited to members of *Cetobacterium*, *Methylobacterium*, *Flavobacterium*, *Methylotenera*, *Hydrogenophaga*, and CK-1C4-19. At day 14, only two OTUs were differentially abundant and by day 21, no OTUs were significantly more abundant when comparing these two classes. Considering the effects of triclosan independent of solvent exposure, at day 14 and day 21, *Hydrogenophaga* OTU624 was increased in relative abundance in high-triclosan exposed samples relative to solvent control samples and, at day 21, *Thauera* OTU37 was increased in relative abundance in high samples relative to solvent samples (Figure 7 and Additional file 10: Table S3 and Additional file 11: Table S4).

### Fathead minnow GI tracts contain highly abundant members of the poorly characterized candidate division CK-1C4-19

**Figure 8:**
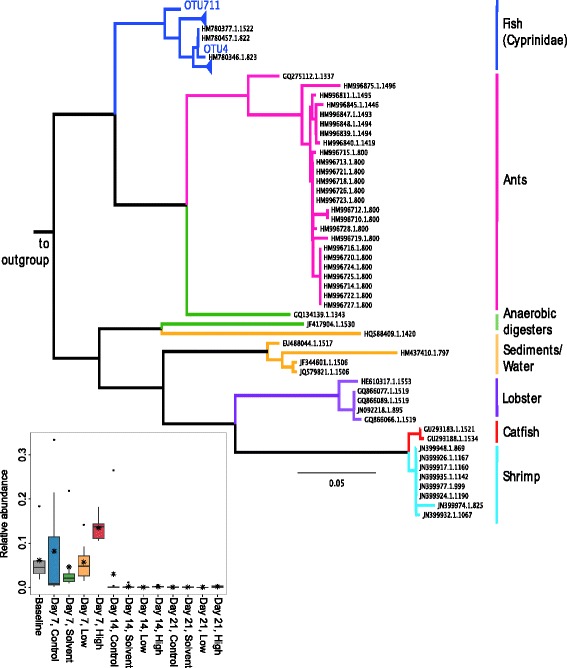
**CK-1C4-19 taxa are phylogenetically distributed by host and habitat.** The V3-V4 regions from publically available 16S rDNA sequences assigned to the candidate division CK-1C4-19 were aligned with CK-1C4-19 sequences found in the fathead minnow gut (OTU4, OTU711), and a phylogenetic tree was constructed. OTUs found in the fathead minnow gut are most closely related to sequences reported from other fish in the family Cyprinidae. *Scale bar* changes per site. Inset: relative abundances by time and treatment of the dominant CK-1C4-19 OTU4.

Within the fathead minnow gut bacterial communities, we identified a highly abundant taxon, CK-1C4-19, that is classified as either a member of the phylum Tenericutes [33] or a candidate division at the phylum level [34]. OTUs from this taxon have been previously identified as members of the zebrafish gut microbiome [23], and a relatively small number of sequences exist in 16S rDNA databases, with annotated environmental and host-associated habitats including other cyprinid fishes, ants, lobster, catfish, sediments, and anaerobic digesters. The two CK-1C4-19 OTUs observed in fathead minnow guts most closely resemble those reported from other cyprinid fishes (Figure 8). This taxon was abundant in all baseline samples, ranging from 2.2% to 22% relative abundance, and was present in all day 7 samples. In general, CK-1C4-19 increased in relative abundance with triclosan exposure, with relative abundance ranging from 1.6% to 14.6% in the day 7 low samples and 10.9% to 18.3% in the day 7 high samples (Figure 8, inset). By comparison, CK-1C4-19 was present as 0.2% to 36% of day 7 control samples and 1.2% to 5.4% of day 7 solvent samples. As this is a change in relative abundance, we cannot rule out that the apparent increase may reflect a decrease in abundance of other taxa, with CK-1C4-19 remaining unchanged. Despite a dramatic drop in abundance after day 7, CK-1C4-19 was present in all but one of the day 14 samples and was found in all but three of the day 21 samples.

## Discussion

Despite the low but environmentally relevant [15] levels (100 to 1,000 ng L^−1^) used here, acute triclosan exposure is sufficient to disrupt fish gut bacterial community structure (Figure 4). In addition, fish microbiomes from the two exposure levels are visually separated in the PCoA, which may indicate a dosage effect. Triclosan targets the final step of the bacterial fatty acid synthesis pathway, inhibiting the active site of the enoyl-acyl reductase FabI [35]. Insensitivity to triclosan results from mutations in the *fabI* gene [35,36], FabI overexpression [35], the expression of alternate Fab proteins such as FabK, FabL, and FabV [37-39], or through efflux [40]. Initially described in *Vibrio* sp*.*, FabV is reported to confer greater resistance to triclosan than mutant forms of FabI [39]. FabV has also been identified in other Gram-negative bacteria including members of the *Aeromonas*, *Shewanella*, and *Pseudomonas* genera, organisms highly abundant in freshwater fish gut microbiomes. The prevalence of fish gut bacterial species maintaining variant Fab genes may explain the stable presence of certain taxa across our study, such as *Aeromonadaceae* and *Shewanella.* In addition, triclosan may be either bactericidal or bacteriostatic depending on concentration [41]. Finally, overall microbial community structure may contribute to differences in triclosan bioavailability and effect, if the community contains members that are resistant to or are able to metabolize triclosan.

Although order-level (Figure 5) or genus-level (Additional file 7: Figure S1) patterns of changes in abundance among exposure groups were clearly visible, many interesting differential responses to triclosan at the OTU-level were obscured by summarizing at higher taxonomic levels. For example, while as an aggregate, all members of the order *Pseudomonadales* are highly abundant over the course of this study (Figure 5); the abundance patterns of the five *Acinetobacter* OTUs in this order suggest differing OTU-level responses to environmental factors, including triclosan (Additional file 12: Figure S8). In addition to *Acinetobacter*, the 26 OTUs whose abundance increased at day 7 in association with triclosan exposure included *Flavobacterium*, *Chryseobacterium*, and *Shewanella*; all four genera contain a subset of species that are documented pathogens of fish and humans [42-45]. Another differentially abundant OTU was classified as candidate division CK-1C4-19, a rarely identified taxon previously documented within the zebrafish gut microbiota [23]. Repeated detection of CK-1C4-19 within the guts of closely related hosts despite geographical distance and its apparent insensitivity to triclosan (Figure 8) warrant further study examining the potential functional contribution to or dependence on its host.

Also included among the OTUs significantly increased in abundance with triclosan exposure were species of *Methylobacterium*, *Hydrogenophaga*, and *Acidovorax* (Figure 6). Methylotrophic proteobacteria such as these are commonly observed as members of denitrifying consortia in wastewater treatment plants [46,47]. These consortia can include *Thauera* and *Methylotenera* [47-49], other genera identified in this study. The microbial denitrification process in wastewater treatment is often facilitated by amendment with carbon substrates including methanol [50] which can be utilized by methylotrophic denitrifiers such as those identified here [51]. Over the course of this study, which used methanol for triclosan solubilization, OTUs identified as *Hydrogenophaga*, *Methylophilaceae*, *Methylotenera*, *Thauera*, and *Acidovorax* displayed a similar pattern of changes in relative abundance (Figure 7 and Additional file 11: Table S4). These OTUs were low in overall abundance at day 7 and became more prominent at later time points, particularly in triclosan-exposed and solvent-exposed samples. This pattern of increase in abundance suggests the possibility that methanol-enabled denitrification can occur within the fish gastrointestinal tract. Alternatively, because we did not measure changes in available nitrogen species in the water or characterize water microbial community changes over time, it is also possible that the combination of methanol (during the 7-day exposure) and the nitrogenous waste from the fish favored the growth of these organisms and that the measured increase in relative abundance of these taxa in the gut may in fact be due to a relative increase in transient bacteria that were sourced from the housing water. While these bacterial taxa were measured from the gut, this study did not differentiate between adherent or transient members of the gut microbial communities.

*Hydrogenophaga* spp. have been identified in association with amphibian hosts [52], but, to our knowledge, *Thauera* sp. have not been reported as members of vertebrate host-associated bacterial communities. *Acidovorax* OTUs have been noted in the trout gut [26] and also within the gut of the marine sea bream in a recent study that speculated on the possibilities of microbial denitrification occurring within the fish gut [53]. The presence of annamox bacterial taxa within fish guts has also been shown within the carp gut [54]. Host-associated denitrification and nitrous oxide production has been studied in freshwater invertebrates [55] and in earthworms [56], which also host *Acidovorax* species [57], but is less explored within vertebrate gut microbial communities, where removal of nitrate via reduction to ammonia rather than via denitrification is noted [58]. Thus, microbial nitrogen cycling pathways within the fish gut may be more diverse than have been reported within mammalian guts. Because of the obvious limitations to attempting to infer function from taxonomic information [59], functional studies are needed, including direct measurement of denitrification-associated gene expression within fish gut microbiomes and housing or habitat waters.

While most studies performed to identify triclosan-degrading bacteria have focused on free-living rather than host-associated organisms, this catabolic ability has been demonstrated in bacteria across a wide variety of taxa [60], and it would not be surprising to find that members of the fish gut microbiome also possess the ability to metabolize or co-metabolize triclosan. Bacterial species related to those identified here have been associated with biodegradation of triclosan or other aromatic or halogenated compounds [47,61-66]. For example, OTU38 is a 100% BLAST match to a fully sequenced strain of *Acidovorax* that degrades poly-chlorinated biphenolic compounds [67], and *Thauera* has been specifically identified as a denitrifying organism with the potential to degrade aromatic compounds [68]. *Thauera* and *Hydrogenophaga* were differentially abundant at day 14 and/or day 21 in high samples with respect to solvent samples (Additional file 10: Table S3), suggesting that these organisms may derive some competitive benefit from even a short exposure to triclosan, independent of the presence of the methanol solvent. Microbial degradation of halogenated aromatics typically requires an aerobic environment [69]. Oxygen availability is likely to be variable over the length of the fish gastrointestinal tract, so sufficient oxygen may be present to support this process. If occurring in the gut, such microbially mediated processes could result in the direct intra-lumenal exposure to triclosan degradation products, including the lipophilic end product methyl-triclosan, which has been shown to bioaccumulate within fish [70].

Broadly, the differences in microbial community structure seen immediately following triclosan exposure (day 7) do not persist during depuration. By the conclusion of 2 weeks of recovery (day 21), as a whole, the communities cannot be distinguished on the basis of prior triclosan exposure, suggesting that most of the gut communities have returned to the same developmental path as those of the unexposed fish. Three samples from the triclosan-exposed groups remained distant from the main group of samples (Figure 4c), and in some cases, restoration of the initial community may take longer than our experimental window permits us to observe, or may never be complete, as has been shown for human microbiomes perturbed with antibiotic exposure [5]. The apparent increase in alpha diversity associated with triclosan exposure (Figure 2) is difficult to interpret, as short-term antibiotic exposure has been shown to decrease [5,6] or increase [6] diversity in other vertebrate guts.

In the case of low concentrations of triclosan, as used here, a bacteriostatic effect on most taxa, rather than a bactericidal one, could result in a near-complete restoration of community structure after depuration; however, even short-term disruption to the gut ecology, as demonstrated here, may be harmful to a developing host, with the potential for both immediate and long-ranging effects. Typical fish commensals such as *Aeromonas*, *Deefgea*, and *Flavobacterium* sp. can also be pathogens [42,71,72] and may be held in check by less abundant, triclosan-sensitive members of the fish gut microbiome [24,73]. Thus, even a brief imbalance from acute exposure may precipitate opportunistic infections [74]. The fish gut microbiome is implicated in nutrient absorption and growth [75,76], so juvenile dysbiosis as a result of environmental toxicants may impact long-term fitness at the individual or population level in contaminated habitats. In surface water ecosystems, increased time (constant rather than acute) and intensity (concentration) of contaminant exposure could easily result in permanent alteration of host microbiome, with potential for ecosystem-scale consequences.

Microbial communities of the fish gut are underexplored relative to the contribution of fish species to overall vertebrate diversity [77]. The fathead minnow (*P. promelas*) is an important model organism for aquatic environmental toxicology [22] and is widely distributed in North America, and its developmental and reproductive response to environmental contaminants is well-characterized [78]. The gut microbiome described here adds to the limited catalog of fish microbiomes characterized by high-throughput sequencing [79]. Despite the temporal and triclosan exposure-associated differences in the microbial community structure, 20 of the 94 OTUs reported here constitute ‘core’ organisms present in 95% of the 103 samples sequenced (Table 2). These study-wide core OTUs include members of *Aeromonadaceae*, *Bacteroidaceae*, *Shewanella*, *Pseudomonas*, *Deefgea*, *Acinetobacter*, *Flavobacterium*, *Cetobacterium*, and others. Both technical differences (cultured vs. sequenced organisms, clones vs. short amplicons, choice of variable region sequenced, primer bias, sequencing depth, analysis) as well as differences related to the fish (age, diet, fresh caught vs. domesticated, water quality, husbandry, habitat) limit the ability to make direct comparisons of relative abundances across studies of other freshwater omnivorous fish gut microbiomes. Despite these caveats, these fathead minnow core microbiome OTUs are generally similar to the most common genera reported previously for zebrafish [23], the common carp [54] (both also family Cyprinidae), other freshwater omnivorous fish [24,25,80], and, in a limited fashion, for the fathead minnow [27]. There are also some differences between the fathead minnow core microbiome and that of other related fishes. For example, the phylum Firmicutes occurs with notable relative abundance in some (but not all) gut communities reported for zebrafish and guppy [25], but is not present in the fathead minnow core microbiome or abundant at any point in the full experiment. Similarity of commensal bacterial communities among phylogenetically related and anatomically similar hosts has been shown for non-fish vertebrate species [81] and suggested for fish [77]. Our study adds to the body of evidence that gut microbial community structure may also be conserved among phylogenetically related fishes.

The gut microbial community of individual fish is likely to be most strongly structured by the interacting effects of 1) host environment and diet, 2) host developmental stage, and 3) triclosan exposure history. The baseline communities were markedly different than days 7 to 21, and this difference made a strong contribution to the overall beta-diversity temporal trajectory (Figure 3a). While almost all OTUs present at days 7 to 21 are also present in the baseline (day 0) samples, because we did not sequence samples of water (autoclaved) or food (commercially prepared), we cannot definitively rule out water or food as a source of the few new OTUs we detected. All exposure groups received food and water from identical stock at each feeding or water change, so any new OTUs that might have arisen from these sources are expected to be evenly distributed across cohorts. The baseline samples were collected immediately upon arrival in our facility, and differing community structure more likely reflects differing conditions in the rearing facility from which the fish were acquired, as diet, water chemistry, and stress have been suggested to be predictive of fish gut microbiome structure [24,25,77,80]. However, even when the highly divergent baseline samples were removed from consideration, communities were in large part structured by time point/developmental stage (Figure 3b). Our study was designed to examine the effect of short-term triclosan exposure on the endogenous gut microbiota of developing, larval fish. Gut microbiomes of developing vertebrates, including fish, are dynamic, showing complex successional processes [82-85]. Thus, the temporally dynamic communities observed here, in addition to effects of triclosan exposure, likely respond to the combined effects of host developmental processes and initial changes to environment.

## Conclusions

Most research on the impact of environmental contaminants has focused on aquatic animals and invertebrates [86] or on water, sediment, and soil microbial communities [51,87]. The effects of environmental contaminants on the host-associated microbiome are largely unexplored. This study demonstrates a shift in the fish gut bacterial community following a 7-day exposure to low, environmentally relevant levels of triclosan. Taxa whose relative abundances change with triclosan exposure include those potentially involved in nitrogen cycling and triclosan metabolism. Even short-duration disruption to the host microbiome such as that shown here may induce long-term effects on the host organism and larger ecosystem.

## Methods

### Experimental design

The effects of acute early-life stage exposure to environmentally relevant concentrations of triclosan on the composition of the gastrointestinal tract microbiome in larval fathead minnow (*P. promelas*) were evaluated in an immersion exposure experiment. Fathead minnow larvae (approximately 8 weeks post-hatch) were obtained from Aquatic Biosystems (Fort Collins, CO). At this developmental stage, fish were sexually undifferentiated or were undergoing differentiation and gender was not determined. The rearing facility classifies fish by hatch age, and the fish in this study were all hatched within a 4-day period. The fish are not isogenic, rather are the progeny of multiple breeding groups. Thus, sibship and age are untested variables in our study.

Eight randomly selected fish were sampled upon arrival as initial controls (baseline; day 0) as described below. The remaining fish were randomly assigned to experimental and control groups. For each group, 15 larvae were placed into each of two 4-L glass jars containing 1 L of solution. All glassware and aeration tubing was autoclaved prior to use, and food was introduced using sterile, single-use serological pipettes. A stock solution of sterile hatched brine shrimp (Hikari Bio-Pure Baby Brine Shrimp, Hayward CA) was prepared daily, and larvae were presented with 2 mL of this stock solution and allowed to feed *ad libitum*. We did not re-verify food sterility in-house or sequence a food-only sample, and thus cannot rule out the possibility that food contributed OTUs to our study; however, at each feeding, all fish were fed from the same stock solution. Our design and methods do not differentiate between transient (including any food-associated) bacteria and adherent bacteria. Test solutions for the 7-day acute exposure were a) control: moderately hard reconstituted water (MHRW) as defined by US Environmental Protection Agency (EPA) protocol #EPA-821-R-02-013 [88], b) solvent control: 0.0001 mg L^−1^ methanol in MHRW, c) low triclosan: 100 ng L^−1^ triclosan in MHRW, and d) high triclosan: 1,000 ng L^−1^ in MHRW. Triclosan (Sigma-Aldrich) was solubilized in methanol, and methanol concentrations were identical across solvent control and triclosan solutions. Methanol was chosen as the solvent for consistency with a recent study focusing on the direct physiological effects of triclosan on the fathead minnow [17]. MHRW involves autoclaved (121°C, 15 psi, 30 min), sterilized water in addition to the additives described in EPA protocol #EPA-821-R-02-013. Our measured pH and alkalinity align with those of similar husbandry schemes and experimental designs, including those of the breeding facility. To utilize environmentally relevant concentrations of triclosan, we chose 100 and 1,000 ng/L which allowed us to work within the Kolpin *et al*. study median of 140 and max of 2,300 ng/L [15], while still working below the reported LC50 for *P. promelas* (260 μg/L at 96-h duration) [89].

Over a 7-day acute exposure, >90% of the test solution was replaced daily. Daily static renewal exchanges consisted of 100 μL aliquots of the pertinent spike aliquot dissolved into 1 L of MHRW. At each daily renewal, every cohort was moved into a freshly autoclaved jar. Following the 7-day acute exposure, larvae from all exposure groups were maintained in control conditions with daily renewals into MHRW and fresh jars until the end of the experiment on day 21 (Figure 1). Photoperiod (14 h light: 10 h dark), temperature (22 ± 1°C), and dissolved oxygen (>85% saturation) did not differ among exposure groups during the 21-day experiment. Animal care and handling was in accordance with the Institutional Animal Care and Use Committee of the University of Colorado Denver, #92514(05)1E.

### Sample collection

Multiple intact gastrointestinal (GI) tracts were collected at four time points for each experimental group: prior to exposure (baseline), immediately after a 7-day exposure (day 7), after 1 week of post-exposure depuration (day 14), and finally at the end of the 21-day experiment (day 21). Fish were anesthetized in ice water prior to euthanasia by rapid decapitation. Body length and mass measurements of anesthetized fish were collected and are included in Additional file 1: Table S1. Freshly dissected GI tracts were placed into filter-sterilized PBS and frozen at −20°C until DNA extraction. Since the gastrointestinal organ is less developed in larval fish compared to adult fish, the transition between esophagus, stomach, and intestines was not distinguishable during dissection. Manipulation instruments were autoclaved, and instruments and surfaces were cleaned with ethanol after each specimen. All work was performed in a biosafety cabinet.

### DNA extraction

Total bacterial and host DNA was extracted using the PowerSoil DNA Isolation Kit (MO BIO Laboratories, Carlsbad CA), with modifications to the standard protocol noted below. Samples were thawed and transferred with 50 μL of PBS storage buffer to the bead tube, which was then vortexed at maximum speed for 10 min to disrupt the intact fish GI tracts. After the addition of solution C1, the tube was heated at 65°C for 10 min, followed by 10 min of vortexing at maximum speed. Following the C3 incubation step, the centrifugation time was increased from 1 to 2 min. Extracted DNA was stored at −20°C. Because these extractions contained varying, unknown ratios of host to microbial gDNA, we used the spectroscopic DNA quantification as rough estimates of extraction success, but were unable to use these measurements for normalization of microbial gDNA mass for downstream processing.

### Marker gene (16S rDNA) amplification and sequencing

Amplicon sequencing of 108 samples targeting the V3-V4 hypervariable regions of the 16S rRNA gene was performed following the dual-indexing strategy of Kozich *et al*. [90]. The 103 experimental samples included eight baseline samples and eight samples per triclosan exposure group for each of the three subsequent time-points (Figure 1), with the exception of ‘day 14 low’ which had only seven samples due to the failure of one sample in DNA extraction. In addition to these 103 samples, we sequenced one technical replicate of sample D0C9, one sample of a mock bacterial community consisting of equal mass of gDNA from ten different bacterial and archaeal species [91] (Table 1), and three mock/spike technical replicates containing a 1:1 combination (by gDNA mass) of the mock community and sample D0C9. D0C9 was chosen because sufficient gDNA was available, and a pilot study suggested that this sample was representative of other baseline samples. PCR reactions included 10 μL Q5 2× Hot Start Master Mix (New England Biolabs, Ipswich MA), 0.5 μL each primer (from 10 μM stock), 1 μL template DNA, and 8 μL nuclease-free water under the following conditions: 30 s at 98°C; 25 cycles of (10 s at 98°C, 15 s at 55°C, 20 s at 72°C); 2 min at 72°C; 4°C hold. Triplicate reactions per sample were combined and cleaned up using the Zymo Clean and Concentrate-5 kit (Zymo Research Corporation, Irvine CA), eluted into 17 μL nuclease-free water and quantified using the Qubit BR dsDNA kit (Life Technologies, Grand Island NY). Additional PCR reactions were performed as needed to generate the 10 ng of cleaned amplicon from each sample included in the pooled sequencing library. Sequencing was performed at the University of Colorado Denver Genomics and Microarray Core with a single lane of Illumina MiSeq using 2 × 251 bp paired end reads and V2 chemistry, with 8% PhiX added to the library. All reads and metadata are deposited in the Sequence Read Archive (SRA; http://www.ncbi.nlm.nih.gov/sra) under BioProject PRJNA257816 (SRA accession SRP045371).

### Read preprocessing, OTU assignment, and OTU filtering

Demultiplexing was performed with CASAVA v. 1.8, and reads representing the PhiX or reads not matching indices were removed. The remaining reads were assigned to OTUs following the UPARSE pipeline (usearch v7.0.1090_i86linux32) [92] with the following non-default parameters: -fastq_mergepairs (-fastq_truncqual 3, -fastq_minmergelen 250); -fastq_filter (-fastq_maxee 1.0, -fastq_truncqual 10, -fastq_minlen 300); -usearch_global (-strand plus, -id 0.97) and custom scripting to accommodate large file sizes. The uchime_ref step was omitted, and the final OTUs were checked for chimeras using the DECIPHER web tool and the short sequences option [93]. Taxonomy was assigned to OTUs in QIIME v 1.8.0 [94], using the RDP classifier v2.2 [95] which was retrained against the Greengenes 13_8 rep set [33] that had been trimmed using PrimerProspector v. 1.0.1 [96] to the V3-V4 region [97]. A phylogenetic tree of the OTU sequences was constructed using FastTree [98].

We used the mock community sample to determine a threshold for filtering our OTU table for spurious or contaminant OTUs. Of the mock community reads, 99.89% were assigned to OTUs representing mock community 16S rRNA sequences. All OTUs in the remaining 0.11% coincided with sequences found in the fish samples, and the most abundant non-mock sequence seen in the mock community sample constituted 0.03% of the reads. We used this value to filter the whole-study OTU table according to the following criteria. To be retained for further analysis, an OTU should be found at greater than 0.03% relative abundance in at least *N* − 3 samples where *N* is the size of a time + exposure group (e.g., day 7, solvent: *N* = 8). This procedure should eliminate or greatly reduce instances of PCR contamination between samples, as we do not expect contamination to occur in a biologically meaningful pattern consistent with our experimental design. Note that OTUs can still occur at <0.03% relative abundance in any given sample. This conservative filtering procedure runs the risk of excluding naturally occurring, low abundance OTUs near the detection limits of the filtering protocol; however, this is not expected to impact our ability to answer the diversity-based question posed in this study regarding the effects of triclosan exposure. We explicitly tested this by using a variety of beta diversity distance metrics on the filtered and unfiltered OTU tables and by calculating alpha diversity on the filtered and unfiltered OTU tables. All analyses presented here are conducted on the filtered OTU table unless otherwise noted, with no additional normalizations or rarefying procedures [99]. Both filtered and unfiltered OTU tables (Additional files 13 and 14) and OTU FASTA files (Additional files 15 and 16) are available.

### CK-1C4-19 phylogeny

Publicly available sequences classified as belonging to the taxon CK-1C4-19 were downloaded from the Silva rRNA database SSU Ref web release 117 [34]. The V3-V4 region was extracted *in silico* from these 124 sequences using PrimerProspector v. 1.0.1 [96]. Of the 124 sequences, 62 contained priming sites deemed likely to successfully amplify. Examination of sequences predicted not to amplify revealed many misclassified eukaryotes or sequences with low pintail scores, which may be chimeras. The remaining 62 sequences were combined with CK-1C4-19 sequences from this study and several *Aeromonas* sequences as an outgroup, aligned using MUSCLE v3.8.31 with default parameters [100], and an approximately-maximum-likelihood phylogenetic tree generated using FastTree 2.1.5 SSE3 [98].

### Statistical analyses

Data visualization and statistical analyses were conducting using QIIME, R (http://www.r-project.org), phyloseq [101], vegan [102], and ggplot2 [103]. Generation of taxa summary bar charts and core microbiome calculation was performed using QIIME v. 1.8.0. All distance measures and ordinations were calculated in R v. 3.1.0 using phyloseq v. 1.8.2, and vegan v. 2.0-10. Shannon’s diversity index was calculated using phyloseq, with ANOVA and Tukey’s HSD tests performed in R. We used PERMANOVA (vegan::adonis) [29] with the weighted UniFrac distance matrix in order to test the ability of multiple variables (time, triclosan exposure) to account for observed variance in inter-sample distances. To calculate the significance of clusters observed in principle coordinates analysis, we used MRPP (vegan::mrpp), a univariate analysis that compares mean within-group distance against the within-group distance of randomly permuted groups [31]. MRPP was performed with 10,000 permutations on a pairwise weighted UniFrac distance matrix for all samples within each time point, with groups defined by triclosan exposure category. To identify differentially abundant OTUs, we used the DESeq2 [32] package for R which has been extended to the analysis of microbial community data via phyloseq [99] with parameters: test = Ward, fit = local, *P* ≤ 0.005.

## Availability of supporting data

The data sets supporting the results of this article are available in the NCBI Sequence Read Archive, BioProject PRJNA257816, SRA accession SRP045371 (http://www.ncbi.nlm.nih.gov/sra).

## Additional files

Additional file 1: Table S1. Sample identification codes, BioSample accession numbers, experiment information, barcoding indices, and number of reads for each of the 108 characterized microbiomes. Additional file 2: Figure S2. Overall patterns of beta-diversity are unaffected by OTU filtering procedure. Principle coordinates analysis of weighted UniFrac inter-sample distances using the unfiltered OTU table differs little from results shown in Figure 3, with samples grouped primarily by time point. Additional file 3: Figure S3. Samples cluster by exposure level immediately following acute triclosan exposure when using the unfiltered OTU table for ordination. Analyses are as in Figure 4. MRPP values: day 7 (*A* = 0.195, *P* < 0.0001); day 14 (*A* = 0.071, *P* = 0.022); day 21 (*A* = 0.003, *P* = 0.377). Additional file 4: Figure S4. Amplicon sequencing of the 16S V3-V4 region is technically reproducible, and filtering removes spurious OTUs. a) Sample D0C9 and its technical replicate are highly similar both pre-filtering for spurious OTUs (Pearson *r* = 0.99) and post-filtering (Pearson *r* = 0.99). b) One mock community sample was prepared and sequenced. Levels of non-mock community OTUs (e.g., from cross-contamination from other fish gut samples) in the mock community sample were used to set a study-wide minimum abundance threshold (≥0.03%) for inclusion. In the full experiment, if an OTU reached this 0.03% threshold in ≥5 of eight samples in any time + exposure group, it was retained for analyses. c) Three spike-in samples were prepared with equal parts mock community gDNA and sample D0C9 gDNA (mixed fish and bacterial community). Because fish gDNA dominates the D0C9 component; the bacterial community identified in pre-filtering spike-in samples is dominated by the mock community, as expected. d) After identifying spurious OTUs from the study-wide filtering, all OTUs from mock community members are successfully removed from the spike-in samples, leaving only OTUs from the D0C9 component. The post-filtering spike-in samples (mean remaining reads per sample = 263) correctly resemble the D0C9 sample (mean pairwise *r* = 0.85). Each color represents a different genus, with taxonomy presented for selected taxa as assigned via the RDP classifier and the Greengenes database (see the ‘Methods’ section). Additional file 5: Figure S5. Relative abundances for the fathead minnow core microbiome OTUs across individual fish. OTUs identified as part of the core microbiome (present across at least 95% of the 103 sample fish) were clustered and ordered by single-linkage hierarchical clustering based on Euclidean distances of day 7 cells. White cells in the heat map indicate no reads assigned to the OTU for that sample. Relative abundances are normalized to the total number of reads per sample, and scale bar (fractional relative abundance) and coloring is identical to Figure 5. Taxonomic assignments of core OTUs are available in Table 2. Columns are B: baseline, C: Control, S: Solvent, L: Low triclosan exposure history, and H: High triclosan exposure history. Additional file 6: Figure S6. Alternate distance and ordination methods do not alter overall study-wide patterns of beta diversity. a) Principle coordinates ordination of Canberra distances for all samples. b) Principle coordinates ordination of Canberra distances with Baseline samples removed. c) NMDS ordination of Morisita distances for all samples. d) NMDS ordination of Morisita distances with Baseline samples removed. Additional file 7: Figure S1. Genus-level diversity presented as relative-abundances per sample of each assigned genus, across all 108 samples referenced in this study. Each color in the stacked bar chart represents a genus. Sample codes are as in Table S1. Additional file 8: Figure S7. Samples cluster by exposure level immediately following acute triclosan exposure when using multiple distance metrics and ordination methods. a to c) Principle coordinates analysis of Canberra distances. MRPP values: day 7 (*A* = 0.0.095, *P* = 0.001); day 14 (*A* = 0.025, *P* = 0.032); day 21 (*A* = 0.011, *P* = 0.124); d to f) NMDS ordination of Morisita distances. MRPP values: day 7 (*A* = 0.0.095, *P* = 0.001); day 14 (*A* = 0.025, *P* = 0.032); day 21 (*A* = 0.011, *P* = 0.124). Analyses are otherwise as in Figure 4, and use the filtered OTU table. Additional file 9: Table S2. DESeq2 results identifying differentially abundant OTUs by triclosan exposure history (high/solvent vs. control/solvent). Twenty-eight OTUs are differentially abundant at day 7, 2 OTUs are differentially abundant at day 14, and no OTUs are differentially abundant at day 21. Additional file 10: Table S3. DESeq2 results identifying differentially abundant OTUs by high triclosan exposure history with respect to methanol solvent-exposed samples (high vs. solvent). Nine OTUs are differentially abundant at day 7, five OTUs are differentially abundant at day 14, and six OTUs are differentially abundant at day 21. Additional file 11: Table S4. Significant pairwise comparisons for OTUs shown in Figure 7. Additional file 12: Figure S8. Abundance patterns of individual OTUs may be obscured by higher-level taxonomic assignment. a) Relative abundance of ten OTUs within order *Pseudomonadales*, half of which are assigned as genus *Acinetobacter.* b to e) Relative abundance of the four *Acinetobacter* OTUs identified as significantly increased in triclosan-exposed samples at day 7. f) *Acinetobacter* OTU34 is not significantly increased in triclosan-exposed at day 7. Additional file 13:
**Filtered OTU table.** Read counts per sample for OTUs passing the filtering procedure, in tab-delimited text format. Additional file 14:
**Unfiltered OTU table.** Read counts per sample for all OTUs, including those not passing the filtering procedure, in tab-delimited text format. Additional file 15:
**FASTA file of filtered OTU sequences.** FASTA formatted text file of DNA sequences for all OTUs passing the filtering procedure. Additional file 16:
**FASTA file of unfiltered OTU sequences.** FASTA formatted text file of DNA sequences for all OTUs, including those not passing the filtering procedure.
