# Supplementary figures and images for: Perturbation and restoration of the fathead minnow gut microbiome after low-level triclosan exposure
Source: Microbiome. 2015 Mar 3;3:6. doi: 10.1186/s40168-015-0069-6 (PMC4374533; doi:10.1186/s40168-015-0069-6)

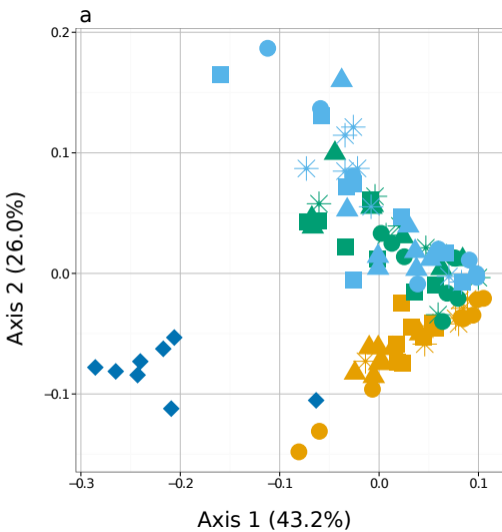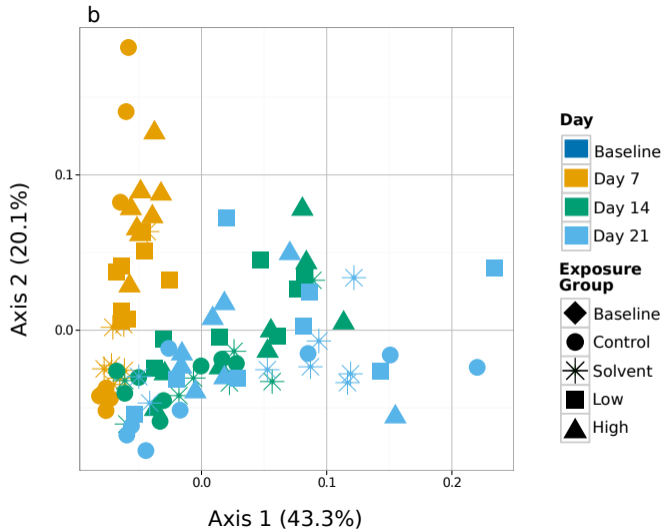

Supplement: Additional file 2: Figure S2. — Overall patterns of beta-diversity are unaffected by OTU filtering procedure. Principle coordinates analysis of weighted UniFrac inter-sample distances using the unfiltered OTU table differs little from results shown in Figure 3, with samples grouped primarily by time point. [file 40168_2015_69_MOESM2_ESM.pdf]

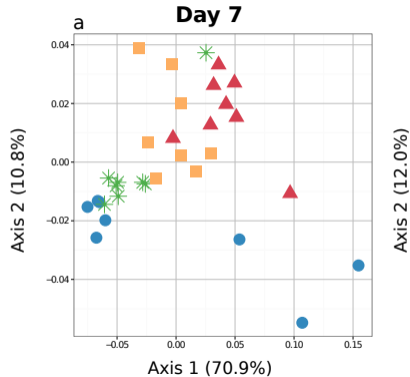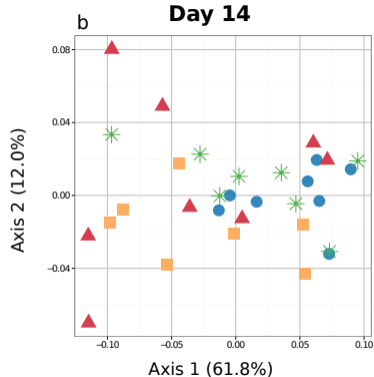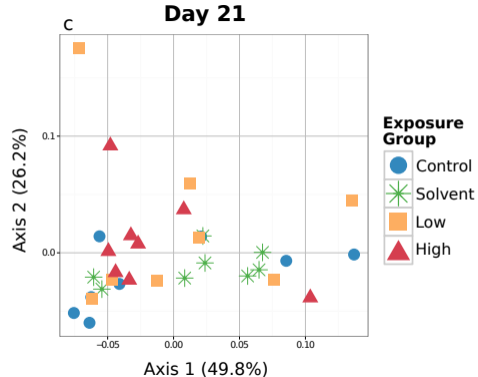

Supplement: Additional file 3: Figure S3. — Samples cluster by exposure level immediately following acute triclosan exposure when using the unfiltered OTU table for ordination. Analyses are as in Figure 4. MRPP values: day 7 (A = 0.195, P < 0.0001); day 14 (A = 0.071, P = 0.022); day 21 (A = 0.003, P = 0.377). [file 40168_2015_69_MOESM3_ESM.pdf]

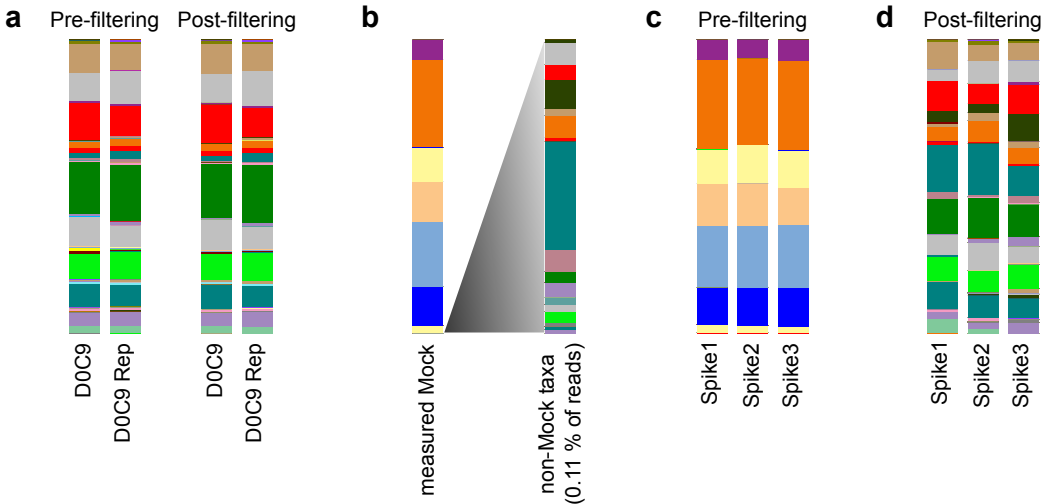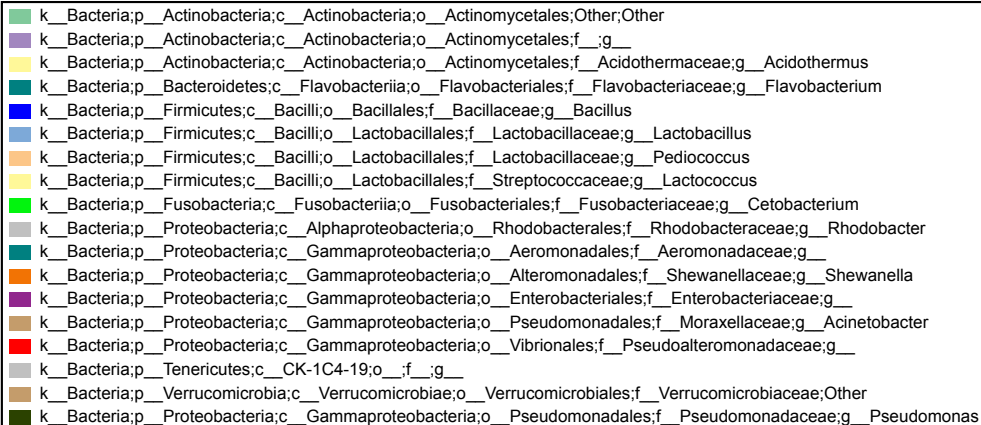

Supplement: Additional file 4: Figure S4. — Amplicon sequencing of the 16S V3-V4 region is technically reproducible, and filtering removes spurious OTUs. a) Sample D0C9 and its technical replicate are highly similar both pre-filtering for spurious OTUs (Pearson r = 0.99) and post-filtering (Pearson r = 0.99). b) One mock community sample was prepared and sequenced. Levels of non-mock community OTUs (e.g., from cross-contamination from other fish gut samples) in the mock community sample were used to set a study-wide minimum abundance threshold (≥0.03%) for inclusion. In the full experiment, if an OTU reached this 0.03% threshold in ≥5 of eight samples in any time + exposure group, it was retained for analyses. c) Three spike-in samples were prepared with equal parts mock community gDNA and sample D0C9 gDNA (mixed fish and bacterial community). Because fish gDNA dominates the D0C9 component; the bacterial community identified in pre-filtering spike-in samples is dominated by the mock community, as expected. d) After identifying spurious OTUs from the study-wide filtering, all OTUs from mock community members are successfully removed from the spike-in samples, leaving only OTUs from the D0C9 component. The post-filtering spike-in samples (mean remaining reads per sample = 263) correctly resemble the D0C9 sample (mean pairwise r = 0.85). Each color represents a different genus, with taxonomy presented for selected taxa as assigned via the RDP classifier and the Greengenes database (see the ‘Methods’ section). [file 40168_2015_69_MOESM4_ESM.pdf]

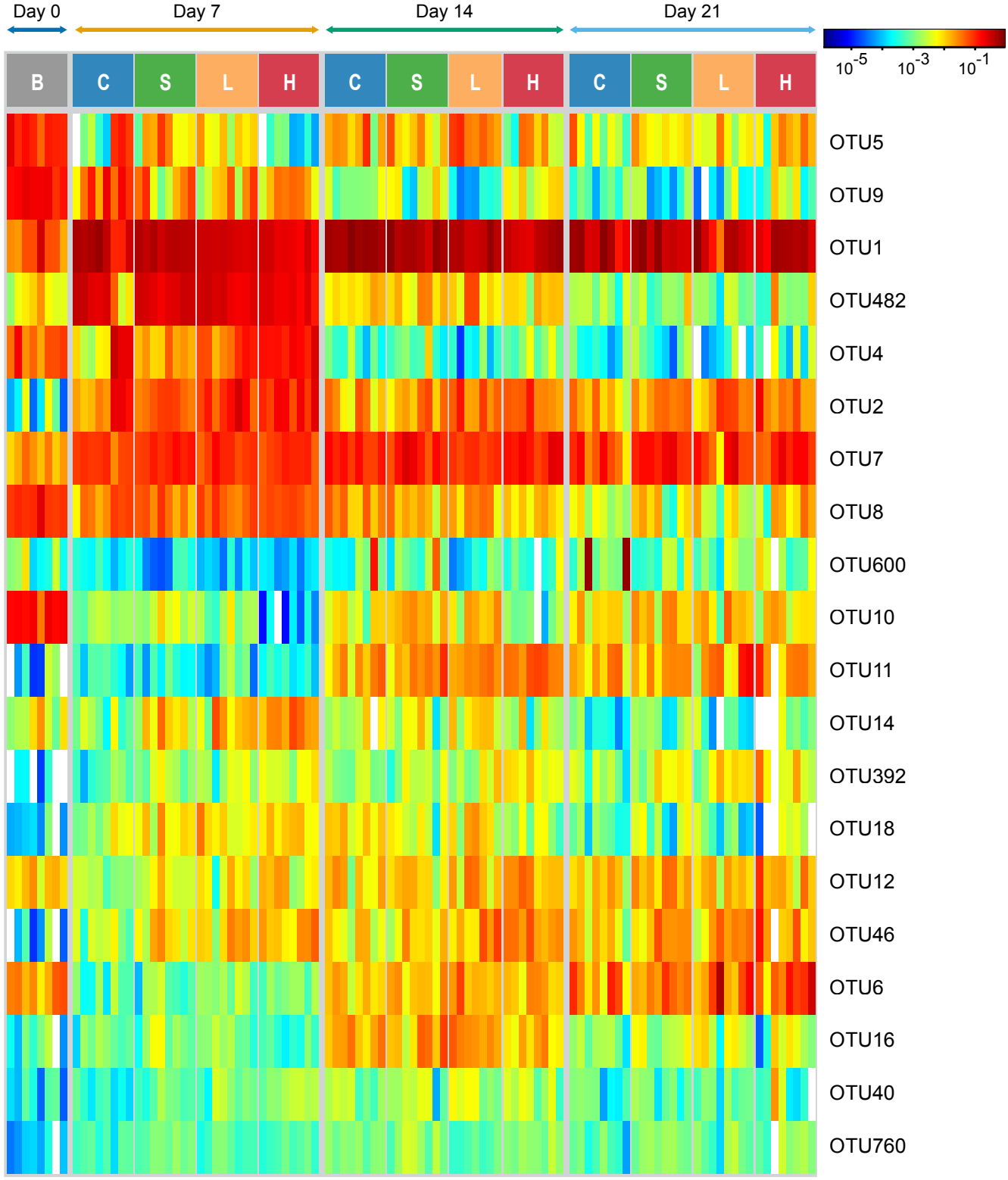

Supplement: Additional file 5: Figure S5. — Relative abundances for the fathead minnow core microbiome OTUs across individual fish. OTUs identified as part of the core microbiome (present across at least 95% of the 103 sample fish) were clustered and ordered by single-linkage hierarchical clustering based on Euclidean distances of day 7 cells. White cells in the heat map indicate no reads assigned to the OTU for that sample. Relative abundances are normalized to the total number of reads per sample, and scale bar (fractional relative abundance) and coloring is identical to Figure 5. Taxonomic assignments of core OTUs are available in Table 2. Columns are B: baseline, C: Control, S: Solvent, L: Low triclosan exposure history, and H: High triclosan exposure history. [file 40168_2015_69_MOESM5_ESM.pdf]

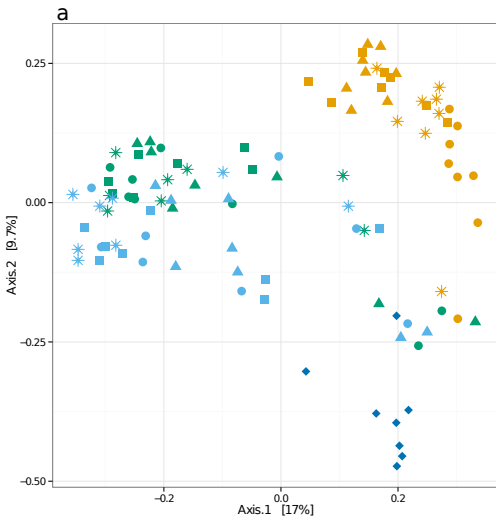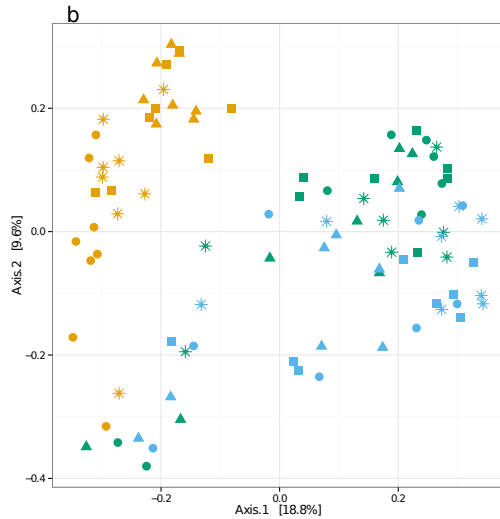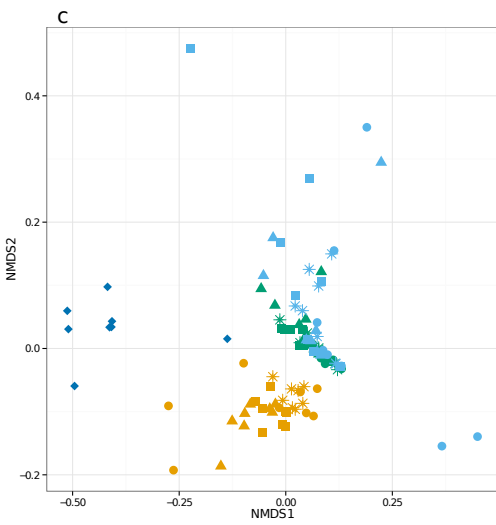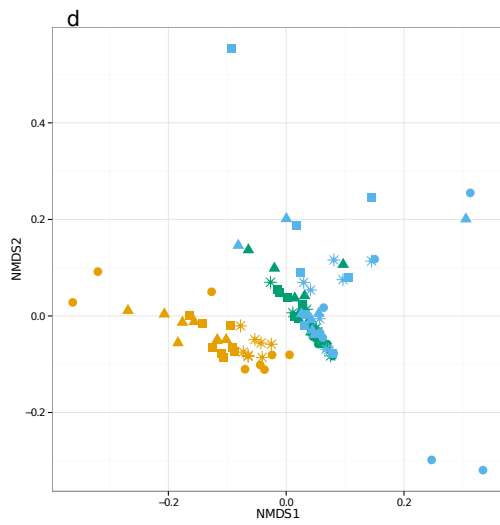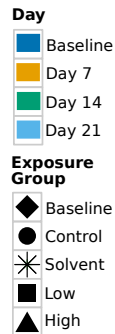

Supplement: Additional file 6: Figure S6. — Alternate distance and ordination methods do not alter overall study-wide patterns of beta diversity. a) Principle coordinates ordination of Canberra distances for all samples. b) Principle coordinates ordination of Canberra distances with Baseline samples removed. c) NMDS ordination of Morisita distances for all samples. d) NMDS ordination of Morisita distances with Baseline samples removed. [file 40168_2015_69_MOESM6_ESM.pdf]

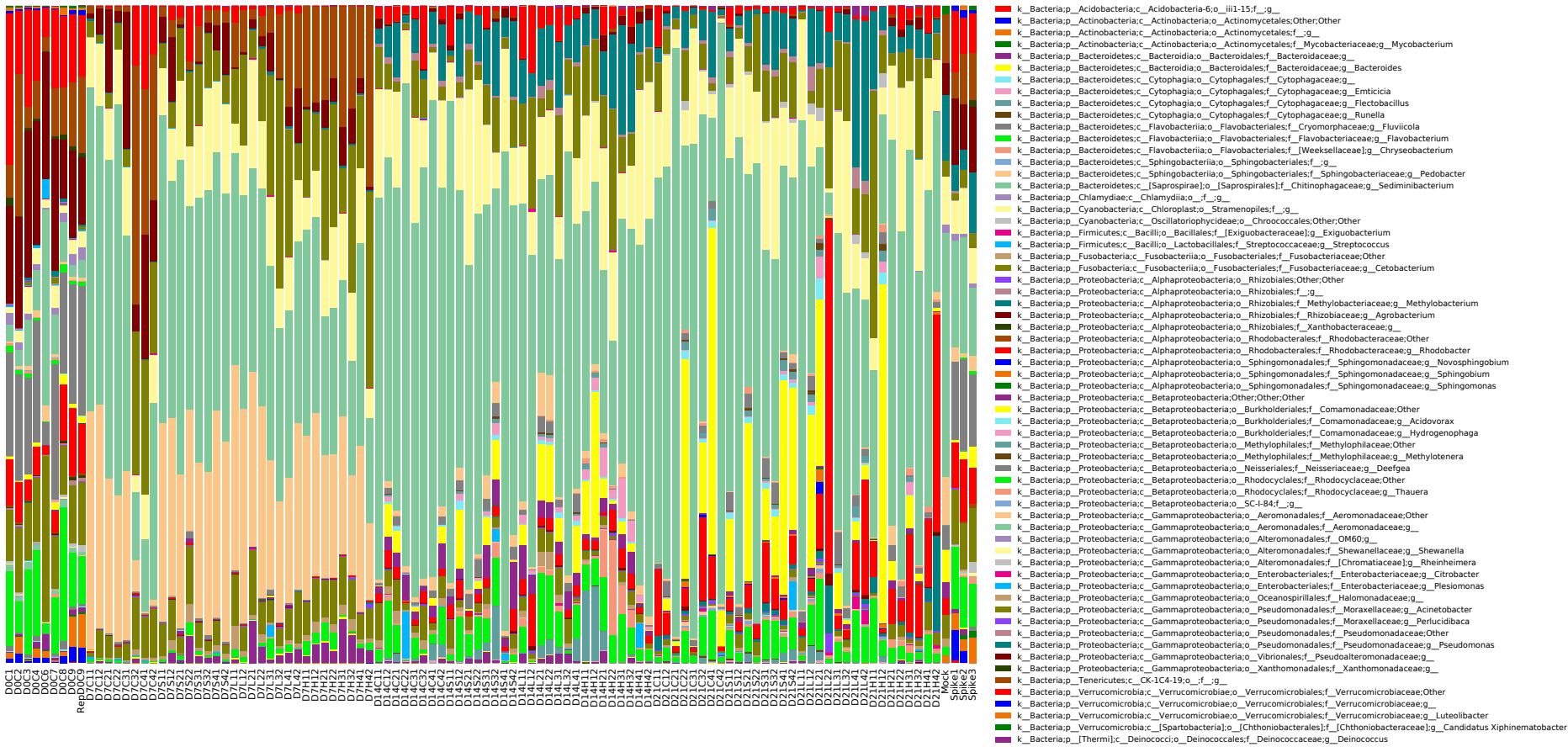

Supplement: Additional file 7: Figure S1. — Genus-level diversity presented as relative-abundances per sample of each assigned genus, across all 108 samples referenced in this study. Each color in the stacked bar chart represents a genus. Sample codes are as in Table S1. [file 40168_2015_69_MOESM7_ESM.pdf]

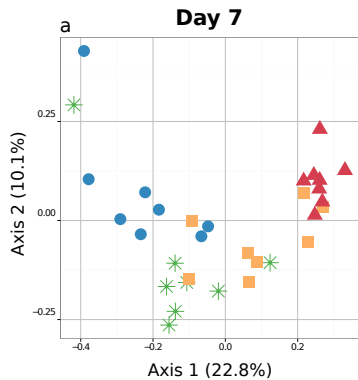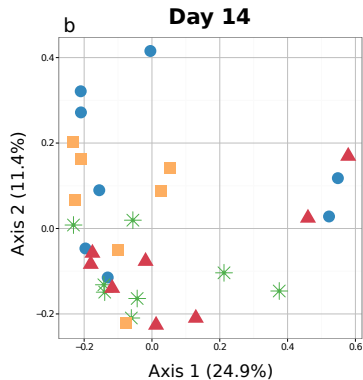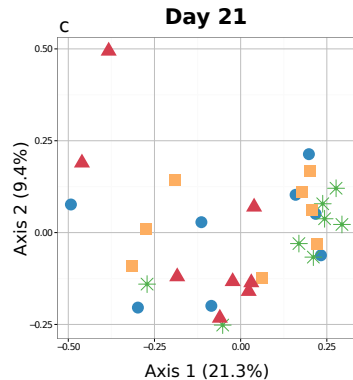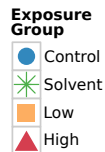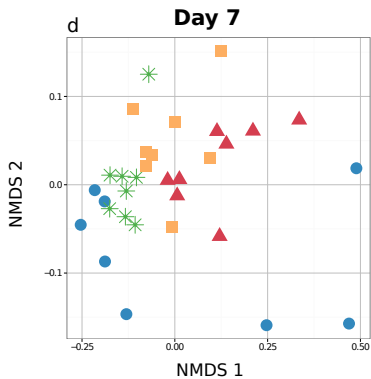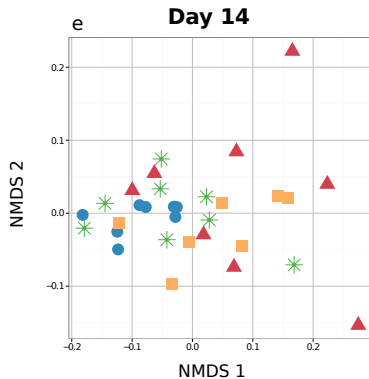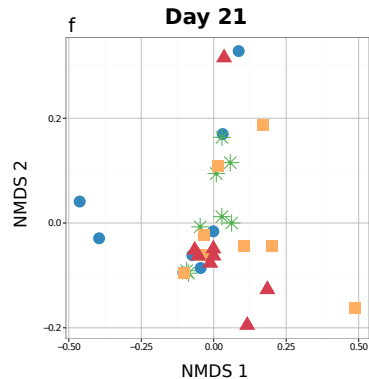

Supplement: Additional file 8: Figure S7. — Samples cluster by exposure level immediately following acute triclosan exposure when using multiple distance metrics and ordination methods. a to c) Principle coordinates analysis of Canberra distances. MRPP values: day 7 (A = 0.0.095, P = 0.001); day 14 (A = 0.025, P = 0.032); day 21 (A = 0.011, P = 0.124); d to f) NMDS ordination of Morisita distances. MRPP values: day 7 (A = 0.0.095, P = 0.001); day 14 (A = 0.025, P = 0.032); day 21 (A = 0.011, P = 0.124). Analyses are otherwise as in Figure 4, and use the filtered OTU table. [file 40168_2015_69_MOESM8_ESM.pdf]

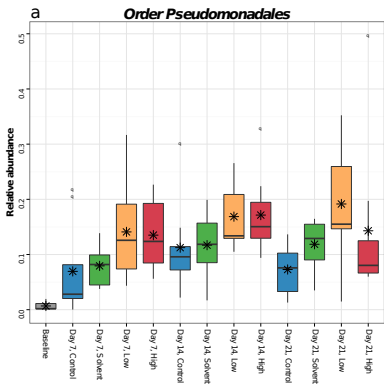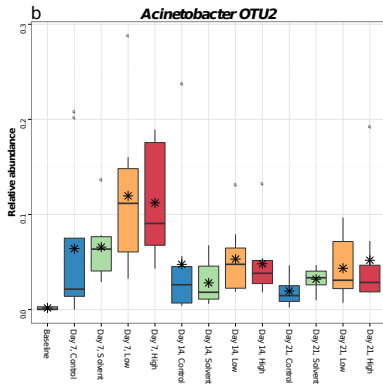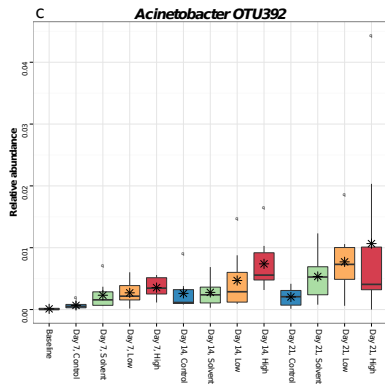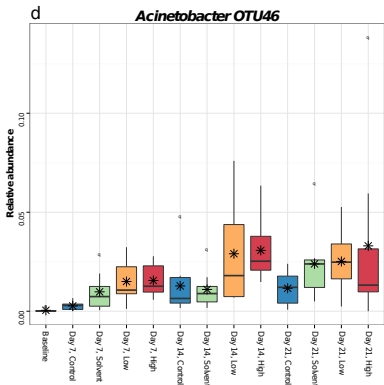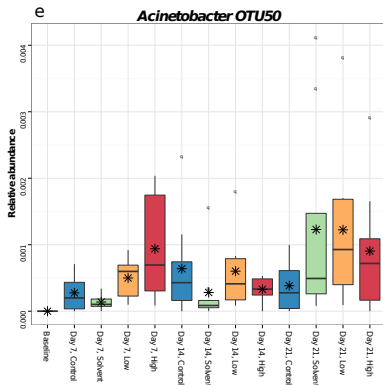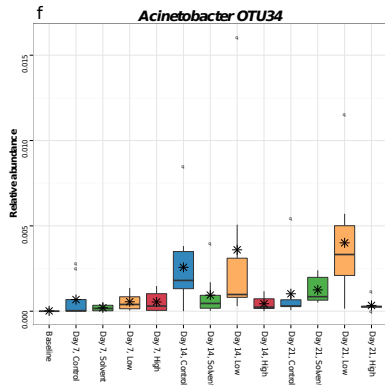

Supplement: Additional file 12: Figure S8. — Abundance patterns of individual OTUs may be obscured by higher-level taxonomic assignment. a) Relative abundance of ten OTUs within order Pseudomonadales, half of which are assigned as genus Acinetobacter. b to e) Relative abundance of the four Acinetobacter OTUs identified as significantly increased in triclosan-exposed samples at day 7. f) Acinetobacter OTU34 is not significantly increased in triclosan-exposed at day 7. [file 40168_2015_69_MOESM12_ESM.pdf]
